# Self-Reported Restrictive Eating, Eating Disorders, Menstrual Dysfunction, and Injuries in Athletes Competing at Different Levels and Sports

**DOI:** 10.3390/nu13093275

**Published:** 2021-09-19

**Authors:** Suvi Ravi, Johanna K. Ihalainen, Ritva S. Taipale-Mikkonen, Urho M. Kujala, Benjamin Waller, Laura Mierlahti, Johanna Lehto, Maarit Valtonen

**Affiliations:** 1 Faculty of Sport and Health Sciences, University of Jyväskylä, 40014 Jyväskylä, Finland; urho.m.kujala@jyu.fi; 2 Neuromuscular Research Center, Biology of Physical Activity, Faculty of Sport and Health Sciences, University of Jyväskylä, 40014 Jyväskylä, Finland; johanna.k.ihalainen@jyu.fi; 3 Sports Technology Unit, Faculty of Sport and Health Sciences, University of Jyväskylä, 88610 Vuokatti, Finland; ritva.s.taipale@jyu.fi; 4 Sport and Health Research Centre, Sports Science Department, School of Social Sciences, Physical Activity, Physical Education, Reykjavik University, 102 Reykjavik, Iceland; ben.waller@jamk.fi; 5 Paavo Nurmi Centre & Unit for Health and Physical Activity, University of Turku, 20520 Turku, Finland; laura.mierlahti@utu.fi; 6 Research Institute for Olympic Sports, 40700 Jyväskylä, Finland; johanna.lehto@kihu.fi (J.L.); maarit.valtonen@kihu.fi (M.V.)

**Keywords:** female athlete, eating disorder, disordered eating, menstrual irregularity, sports injury

## Abstract

The purpose of this study was to investigate the prevalence of self-reported restrictive eating, current or past eating disorder, and menstrual dysfunction and their relationships with injuries. Furthermore, we aimed to compare these prevalences and associations between younger (aged 15–24) and older (aged 25–45) athletes, between elite and non-elite athletes, and between athletes competing in lean and non-lean sports. Data were collected using a web-based questionnaire. Participants were 846 female athletes representing 67 different sports. Results showed that 25%, 18%, and 32% of the athletes reported restrictive eating, eating disorders, and menstrual dysfunction, respectively. Higher rates of lean sport athletes compared with non-lean sport athletes reported these symptoms, while no differences were found between elite and non-elite athletes. Younger athletes reported higher rates of menstrual dysfunction and lower lifetime prevalence of eating disorders. Both restrictive eating (OR 1.41, 95% CI 1.02–1.94) and eating disorders (OR 1.89, 95% CI 1.31–2.73) were associated with injuries, while menstrual dysfunction was associated with more missed participation days compared with a regular menstrual cycle (OR 1.79, 95% CI 1.05–3.07). Our findings indicate that eating disorder symptoms and menstrual dysfunction are common problems in athletes that should be managed properly as they are linked to injuries and missed training/competition days.

## 1. Introduction

Athletes may feel pressure to alter their weight in order to improve performance or subjective appearance, which can lead to dieting, restrictive and disordered eating, or even a clinical eating disorder [1]. Indeed, attitudes toward eating and body image can be seen as a continuum from energy balance and healthy body image to disordered eating, culminating with a clinical eating disorder, such as anorexia nervosa or bulimia nervosa [2]. Disordered eating is defined as abnormal eating behaviors, including, for example, restrictive eating, fasting, skipping meals, and vomiting [3]. Clinical eating disorders and disordered eating, especially restrictive eating, are often associated with low energy availability, which may consequently result in suppression of the hypothalamus–pituitary–ovarian axis and further lead to menstrual dysfunction [3].

Disordered eating, eating disorders, and menstrual dysfunction are common among female athletes [4,5,6]. For example, De Souza and colleagues [5] reported that the prevalence of disordered eating in female collegiate and elite athletes varies from 6% to 42%, while eating disorder prevalence among female athletes ranges from 2% to 33% [4]. Furthermore, the prevalence of menstrual dysfunction in female athletes is reported to vary from 9% to 60% [6]. Meanwhile, the prevalence of disordered eating, eating disorders, and menstrual dysfunction appears to depend on the type of sport, with athletes competing in sports emphasizing leanness being at higher risk than non-lean sport athletes [3,7,8]. Relatively few studies have, however, investigated if the level of sport participation is linked to disordered eating or menstrual dysfunction prevalence among athletes. Existing evidence suggests that elite athletes exhibit higher levels of eating disorder symptoms compared to non-elite athletes or to physically active females that are not competing [9].

Participation in competitive sport is associated with multiple health benefits but also with injury risk [10]. Injuries have been found to be more common in adolescent athletes with disordered eating than among those with healthy attitudes toward body image and eating [11,12,13]. For example, Thein-Nissenbaum et al. [12] reported that athletes with disordered eating had an odds ratio (95% confidence interval) of 2.3 (1.4–4.0) for an injury (overuse and traumatic injuries combined) compared to athletes with no disordered eating. Furthermore, Scheid and Stefanik [11] found that athletes with a high drive for thinness (i.e., disordered eating) had a 69% increase in the number of injuries compared with those reporting a low drive for thinness. However, few studies have investigated the issue in athletes above adolescent age. Recently, Ackerman et al. [14] found no relationship between disordered eating and injury rate in 15–30 year old athletes (mean 18.9 years, SD ± 3.3), although endurance athletes with disordered eating (assessed by questionnaires) trended toward higher injury rates than endurance athletes without disordered eating.

Studies investigating menstrual dysfunction and musculoskeletal injuries in athletes have produced conflicting results [12,15,16,17,18,19,20]. While Rauh et al. [17] found that athletes with menstrual dysfunction had higher odds of injuries (OR 2.9, 95% CI 1.4–6.1) than eumenorrheic athletes, other studies have found no association between menstrual dysfunction and injuries [12,15,16,19]. In some cases, menstrual dysfunction has even been associated with fewer injuries, as von Rosen et al. [20] found that athletes with menstrual dysfunction had fewer current injuries than those with a regular menstrual cycle (21.9% vs. 38%, *p* = 0.024).

To understand the role of the participation level, age, and type of sport on eating problems and menstrual dysfunction, and to evaluate their relationships with injuries, the aim of this study was twofold. First, we aimed to determine the prevalence of self-reported (i) restrictive eating, (ii) current or past eating disorder, and (iii) menstrual dysfunction in a group of female athletes, and to compare the prevalence of these issues in (i) elite and non-elite athletes, (ii) younger and older athletes, and (iii) lean and non-lean sport athletes. Secondly, our purpose was to investigate if there is a relationship of (i) restrictive eating, (ii) current or past eating disorder, and/or (iii) menstrual dysfunction with injuries in this group of athletes and to explore if these associations are different (i) in elite and non-elite athletes, (ii) in younger and older athletes, or (iii) in athletes competing in lean and non-lean sports.

## 2. Materials and Methods

### 2.1. Study Design

The current study was part of a larger cross-sectional survey-based study implemented using an online questionnaire constructed with Webropol 3.0 Online Survey and Reporting Tool (Webropol Oy 2020, Helsinki, Finland). The questionnaire gathered data from six areas: (i) participant characteristics, (ii) low energy availability-related issues (i.e., injuries, gastrointestinal function, and menstrual cycle) using the Low Energy Availability in Females Questionnaire (LEAF-Q) [21], (iii) disordered eating (including restrictive eating) and eating disorders using the female athlete triad screening questionnaire [22], (iv) menstrual cycle function and hormonal contraception, (v) athletes’ perception of the effects of menstrual cycle on performance, and (vi) communication around menstrual cycle. This study primarily used data on disordered eating/eating disorders, menstrual function, and injuries. The questionnaire was tested in a small group of athletes before data collection, and only minor changes (i.e., changes in the order of the questions and a few misspellings) were made on the basis of their feedback.

### 2.2. Recruitment Strategy and Participants

A link to the questionnaire was promoted by the Finnish Olympic Committee, national sports federations, and sports academies, and it was also distributed via social media (e.g., Twitter, Instagram). The survey was available between May 2020 and August 2020.

All female athletes at least 15 years of age were eligible for participation in the study, regardless of the participation level. The questionnaire was offered only in Finnish. The study was evaluated by the Ethical Committee of University of Jyväskylä. Before filling in the questionnaire, participants were informed about the aims of the study and the content of the questionnaire. They were informed that participation was voluntary and that they were free to drop out or leave questions unanswered at any point. No personal identification information was collected; however, participants were given the opportunity to provide their email address in order to give permission for further contact by the research group.

Initially, 926 athletes filled in the questionnaire. Thirty-two questionnaires were excluded due to incomplete data (responders answered only questions concerning menstrual function). For the purpose of the current study, all athletes over 45 years of age (*n* = 23) or those who did not report their age (*n* = 9) were excluded in an attempt to include only premenopausal women. In addition, those who did not provide information on their sports discipline (*n* = 7) or participation level (*n* = 9) were excluded. The final sample size of the current study was 846. Participants represented 67 different sports (Table S1). 

### 2.3. Data Collection

Participants reported their participation level by choosing one of the following options: recreational athlete, regional/district-level athlete, national-level athlete, or international-level athlete. Recreational athletes and athletes competing at regional/district level were assigned into the non-elite athletes group and those competing at national or international level composed the elite athletes group. 

Participants self-reported their year of birth, and their age was calculated as 2020 minus the year of birth. Participants were classified as younger (15–24 years old) and older athletes (25–45 years old).

Participants reported their main sports and were categorized into lean sport athletes and non-lean sport athletes. Lean sport athletes competed in sports where leanness or weight are considered important (endurance, aesthetic, weight class, and antigravitation sports) and non-lean sport athletes in sports where these factors are not considered to have a great impact on performance (ball games, as well as technical and power sports) [8]. For details of this sports type classification, see Table S1.

Participants’ BMI was calculated from reported height and weight. In addition, participants reported their training volume as training hours in the preceding year.

Questions from the female athlete triad screening questionnaire (Mountjoy et al., 2015) were utilized when assessing restrictive eating and eating disorders. Participants were assigned into the restrictive eating group if they answered “yes” to the following question: “Do you limit or carefully control the foods that you eat?” They were classified into the eating disorder group if they answered “yes” to the following question: “Do you currently or have you ever suffered from an eating disorder?” 

Questions from the LEAF-Q [21], as well as questions designed by our own research group, were utilized as means to assess menstrual function and hormonal contraceptive use. A participant was classified into the menstrual dysfunction group if her menstrual cycle was >35 days, she had not had periods in the preceding three months, she had had <9 periods in the preceding year, she had not yet reached menarche despite being ≥15 years old (primary amenorrhea) [3,23], or she reported using hormonal contraceptives because otherwise menstruation stops [24]. Participants using some form of hormonal contraceptives (oral contraceptives, implants, injections, transdermal patches, vaginal rings, or intrauterine systems) for any other reason (contraception, reduction in menstruation pains, reduction in bleeding, to regulate the menstrual cycle in relation to performance, etc.) were excluded from the analysis regarding menstrual dysfunction (*n* = 344).

Regarding injuries, participants were asked if they had had absences from training or competitions during the preceding year due to sports injuries and were classified as injured and non-injured athletes on the basis of their responses. Injured athletes were asked to report how many training or competition days they had missed in the preceding year due to injuries from the following options: 1–7 days, 8–14 days, 15–21 days, or 22 days or more [21]. Participants were assigned into two groups on the basis of their responses: those who had missed ≥22 and those who had missed <22 participation days. In the current study, the definition of injury included both acute and overuse injuries. 

### 2.4. Statistical Analysis

The continuous variables were tested for normality prior to statistical analysis. Descriptive statistics are presented as means and standard deviations (SD) for normally distributed data, medians and interquartile ranges (IQR) for non-normally distributed data, and percentages and counts for categorical data. Differences in prevalences between groups were analyzed using chi-square or Fisher’s exact tests for categorical variables and two-independent-sample *t*-test or Mann–Whitney U test for continuous variables. Logistic regression analysis was used to determine crude and adjusted odds ratios (ORs) and their 95% confidence intervals (95% CIs) for the association of restrictive eating, eating disorders, and menstrual dysfunction with injury occurrence and missed participation days. Statistical analyses were conducted using IBM SPSS Statistics version 24 (Armonk, NY, USA). The significance level was set at *p* < 0.05, two-tailed.

## 3. Results

### 3.1. Prevalence of Restrictive Eating, Eating Disorders, and Menstrual Dysfunction in Different Groups of Athletes

The proportion of athletes reporting restrictive eating, current or past eating disorder, menstrual dysfunction, primary amenorrhea, injuries, and ≥22 missed participation days are presented in Table 1. No differences in any of the abovementioned variables between elite and non-elite athletes were found. Younger athletes reported lower rates of current or previous eating disorders than older athletes, while the prevalence of menstrual dysfunction was lower in the older athletes compared with the younger. Lean sport athletes reported more restrictive eating, eating disorders, and menstrual dysfunction than non-lean sport athletes; however, more non-lean sport athletes had sustained at least one injury during the preceding year than lean sport athletes. No differences were found in current or past primary amenorrhea between elite and non-elite athletes or between lean and non-lean athletes, but there was a trend toward higher rates of primary amenorrhea in elite athletes compared with non-elite athletes (*p* = 0.08) and in lean sport athletes compared with non-lean sport athletes (*p* = 0.06) (Table 1). 

Athletes reporting restrictive eating did not differ from those not reporting restrictive eating in terms of BMI (median 22.5 kg/m^2^ (IQR 20.6–24.8) vs. 22.6 kg/m^2^ (IQR 21.0–24.3), respectively, *p* = 0.741) or training volume (median 600 (IQR 364–800) vs. 520 (IQR 390–720), respectively *p* = 0.092). Athletes reporting a current or past eating disorder had similar BMI (median 22.3 kg/m^2^ (IQR 20.6–24.1) vs. 22.6 kg/m^2^ (IQR 21.0–24.4), respectively, *p* = 0.198) and training hours (median 555 (IQR 366–726) vs. 540 (IQR 400–730), respectively, *p* = 0.983) to those reporting no eating disorder. Athletes reporting menstrual dysfunction had lower BMI (median 21.6 kg/m^2^ (IQR 20.1–23.6) vs. 22.7 kg/m^2^ (IQR 21.0–24.3), *p* < 0.001) and higher training volume (median 627 h (IQR 414–832) vs. 550 h (IQR 400–720), *p* = 0.041) than athletes reporting regular menstrual cycles. For additional descriptive data of the participants, see Table S2.

### 3.2. Restrictive Eating, Eating Disorders, Menstrual Dysfunction, and Injuries

Odds ratios for injury occurrence and ≥22 missed participation days by restrictive eating, eating disorders, and menstrual dysfunction among all participants are presented in Table 2. Athletes with restrictive eating or current or past eating disorder were more likely to report an injury than those with no restrictive eating or eating disorder. No differences were found in injury occurrence among athletes by the menstrual status, or in missed participation days by restrictive eating or eating disorder. However, athletes reporting menstrual dysfunction were more likely to report ≥22 missed participation days than those reporting regular menstrual cycles.

The association between restrictive eating and injury occurrence was seen in non-elite athletes (OR 1.90, 95% CI 1.01–3.57) and in lean sport athletes (OR 1.68, 95% CI 1.14–2.47, but not in elite, non-lean, younger, and older athletes. The association between eating disorder and injuries was observed in elite (OR 1.91, 95% CI 1.24–2.93) and non-elite athletes (OR 2.41, 95% CI 1.11–5.23), in older athletes (OR 2.46, 95% CI 1.44–4.19), and in lean (OR 1.91, 95% CI 1.24–2.93) and non-lean athletes (OR 2.41, 95% CI 1.11–5.23). The relationship between menstrual dysfunction and ≥22 missed participation days was observed in elite (OR 2.37, 95% CI 1.28–4.36) and lean sport athletes (OR 2.07, 95% CI 1.05–4.11).

The relationships of restrictive eating and eating disorder with one or more injuries during the preceding year among all participants still existed when adjusted for BMI, training hours in the preceding year, and age. However, no association between menstrual dysfunction and missed participation days among all athletes was found when adjusting for the abovementioned confounders (Table 3).

## 4. Discussion

The main findings of this large survey-based study were as follows: (1) 25%, 18%, and 32% of the athletes reported restrictive eating, current or past eating disorder, and menstrual dysfunction, respectively; (2) lean sport athletes had higher rates of restrictive eating, eating disorders, and menstrual dysfunction than non-lean sport athletes, while no differences in any of these variables were seen between elite and non-elite athletes; (3) younger athletes reported higher rates of menstrual dysfunction and lower rates of current or past eating disorders than older athletes; (4) restrictive eating and eating disorders were associated with injury occurrence; (5) athletes reporting menstrual dysfunction were more likely to report more missed participation days due to injuries than athletes reporting regular menstrual cycles.

### 4.1. Prevalence of Restrictive Eating, Eating Disorders, and Menstrual Dysfunction

In the present study, 24.6% of the athletes reported restrictive eating, which is higher than the 11.1% reported by Hinton and Beck [25] in female collegiate athletes, but lower than the 39.6% reported by Bennell et al. [26] in female 17–26 year old track-and-field athletes. The lifetime prevalence of self-reported eating disorder in our sample was 18.1%, which is similar to the 17.9% reported by Silén et al. [27] in a Finnish female community-based sample and slightly higher than reports in French athletes (11.2%) [28]. However, the lifetime prevalence of eating disorder observed in this study was lower than self-reported current eating disorder in a sample of Norwegian athletes (27.3%) [29]. Menstrual dysfunction prevalence in the current study among all participants was 31.6%, which is lower than what we recently found in Finnish athletes aged 18–20 years (38.9%) [30], but higher than what was found in high-school athletes (18.8–20.1%) [12,31] and in Finnish athletes aged 14–16 years (17.7%) [30]. The prevalence of a current or past primary amenorrhea in the present study among all participants was 15.5%, which is higher than reported earlier [8,30]. Differences in study populations and methodologies are likely to explain the discrepancies between studies.

In the present study, there were no differences in restrictive eating or eating disorder prevalence between elite and non-elite athletes, i.e., in those competing at national or international level vs. in those competing at regional/district level, which contrasts with the findings of Kong and Harris [9] who found that elite athletes (athletes competing at international or national level and training at least 12 h per week) had higher levels of eating disorder symptoms than noncompetitive athletes or those competing at local to national level and training less than 12 h per week. The differences between our findings and those of Kong and Harris [9] may be attributed to, as with the whole sample, different methodologies and/or study populations. Our findings indicate that, in addition to elite athletes, athletes competing at lower levels are also at high risk for eating disorder symptoms.

While we did not find any relationship between menstrual dysfunction prevalence and the participation level, higher training volume was associated with menstrual dysfunction. This finding is consistent with previous studies [16,32]. However, not all studies have found this association [8]. We recently showed that self-reported weekly training volume was not associated with menstrual dysfunction in athletes aged 18–20 years; however, athletes reporting menstrual dysfunction exhibited higher rates of accelerometer-measured moderate-to-vigorous physical activity than athletes that reported regular menstrual cycles. This association was not, however, observed in athletes aged 14–16 years [30]. The results of the present study indicate that it is not the participation level per se that is associated with menstrual dysfunction, but training volume. Higher training hours may lead to low energy availability, which has been shown to cause alterations in the hypothalamic–pituitary–ovarian axis, which may lead to menstrual dysfunction [3,33].

The older athletes in our sample reported higher rates of current or past eating disorders than the younger ones, while no differences in restrictive eating between the age groups were found. We did not ask participants at what age they received the diagnosis, nor did we differentiate between current and past eating disorders. Thus, the prevalence of a current eating disorder is not known for either group. In earlier studies, the incidence of eating disorders was shown to be highest in adolescence and early adulthood [34].

In the present study, younger athletes reported higher rates of menstrual dysfunction than older athletes, which contradicts the findings of Torstveit and Sundgot-Borgen [8], who found no difference in menstrual dysfunction prevalence in athletes or controls divided into three age groups (16–19, 20–29, and 30–39 years). However, it has been suggested that the risk of menstrual dysfunction decreases with advanced gynecological age (chronological age minus age at menarche) [35], which might explain our findings.

Our results are consistent with a study conducted by Torstveit et al. [29] showing that eating disorder prevalence is higher in lean sport athletes compared with non-lean sport athletes. In addition, eating disorder symptoms, including restrictive eating, have been shown to be higher in lean sport athletes compared with non-lean sport athletes [7,36,37,38], which is in accordance with our findings. Lean sport athletes are reported to have higher desire and external social pressures to be thin, as well as higher rates of body dissatisfaction than non-lean sport athletes [37,38], which may explain these findings. Furthermore, we found that menstrual dysfunction was more common in lean sport athletes compared with non-lean sport athletes, which is consistent with previous studies [8,31,39]. Lean sport athletes might be more susceptible to dieting and unhealthy body image than non-lean sport athletes as they may feel more pressures to maintain a low weight and fat percentage for aesthetic, performance, or sociocultural reasons [1] and, thus, may suffer from low energy availability and also menstrual dysfunction.

### 4.2. Restrictive Eating, Eating Disorders, and Injuries

In the current study, both restrictive eating and eating disorders were associated with injuries, which is in accordance with previous findings [11,12,13]. While previous studies have been conducted in adolescent athletes [11,12,13], our study showed that the association between eating disorders and injuries applies also in older athletes, which suggests that increasing age does not protect athletes from injuries associated with restrictive eating and eating disorders. With respect to restrictive eating and injuries in older athletes, as well as both restrictive eating and eating disorders and injuries in younger athletes, we may have lacked statistical power to reveal significant differences between the groups. Because we did not ask the participants if they are still suffering from an eating disorder or have already recovered from it, we cannot determine if a previous eating disorder was associated with the risk of injuries in these athletes.

### 4.3. Menstrual Dysfunction and Injuries

We found no relationship between menstrual dysfunction and injury occurrence, which is in contrast with the studies conducted by Rauh et al. [17,18] but in agreement with some other studies [12,15,16,19]. However, in the present study, menstrual dysfunction was associated with more missed participation days, which is in accordance with studies conducted by Beckvid Henriksson et al. [16] and Ihalainen et al. [40]. It is known that menstrual dysfunction often results from low energy availability in athletes [3]. Indeed, athletes with menstrual dysfunction in the present study had lower BMI, although median BMI was still within the normal range (i.e., 21.6 kg/m^2^), and they trained more than participants reporting regular menstrual cycles. Thus, it is possible that athletes with menstrual dysfunction in the present study might have had lower energy availability than those with regular cycles. Furthermore, the association between menstrual dysfunction and more missed participation days was observed in elite and lean sport athletes who also trained more and were leaner than non-elite and non-lean sport athletes. This and the fact that no association between menstrual dysfunction and missed participation days was found when adjusting for BMI and training hours might indicate that BMI and training hours mediate the association between those variables.

### 4.4. Limitations

Our study was not without limitations. First, as this was a cross-sectional study, any causal inferences could not be made. Second, we used self-reported data instead of clinical examination, whereas nuance and details regarding restrictive eating and eating disorders were not examined. Third, there was a possibility of self-selection bias, which could have affected our findings. Fourth, we were not able to provide a response rate as we used an open survey, and it is not known how many athletes were reached. Lastly, our study did not include a non-athletic control group and, thus, we could not compare the prevalence of restrictive eating, eating disorders, and menstrual dysfunction in athletes to nonathletes. It should also be noted that the data were collected during the COVID-19 pandemic and restrictions. We cannot exclude the possibility that this influenced some of the answers given by the participants. Despite these limitations, our large sample size and utilization of validated questionnaires enabled us to show the prevalences of restrictive eating, eating disorders, and menstrual dysfunction and their relationships with injuries in different-aged Finnish athletes competing at different levels and sports.

## 5. Conclusions

The prevalence of self-reported restrictive eating, current or previous eating disorder, and menstrual dysfunction in a sample of 15–45 years old Finnish elite and non-elite athletes was 25%, 18%, and 32%, respectively. Lean sport athletes reported higher levels of those symptoms than non-lean sport athletes, while younger athletes (aged 15–24 years) exhibited lower prevalence of current or previous eating disorder and higher prevalence of menstrual dysfunction than older athletes (aged 25–45 years). We found no differences between elite and non-elite athletes regarding restrictive eating, eating disorders, or menstrual dysfunction. Restrictive eating and current or past eating disorder were associated with injury occurrence, and athletes with menstrual dysfunction reported more missed participation days due to injuries than regularly menstruating athletes. Athletes and those working with them should understand the role of eating disorder symptoms and menstrual dysfunction in injury risk, in addition to other consequences for health and performance that have been associated with eating disorders/disordered eating and menstrual dysfunction [3,41]. Eating disorder symptoms and menstrual dysfunction should be taken seriously, and athletes presenting with these symptoms should receive medical evaluation and appropriate treatment. In addition, an athlete’s readiness to participate in sports should be evaluated [41]. Our results can also motivate athletes to seek help for problems with eating and menstrual cycle as our findings indicate that these problems are associated with more injuries/fewer training days.

## Figures and Tables

**Table 1:** Proportion of athletes reporting restrictive eating (RE), current or past eating disorder (ED), menstrual dysfunction (MD), current or past primary amenorrhea (PA), one or more injuries in the preceding year, and ≥22 missed participation days (from training or competition) due to injuries.

	All Participants	Non-Elite Athletes	Elite Athletes	*p*-Value	Odds Ratio (95% CI)	Younger Athletes	Older Athletes	*p*-Value	Odds Ratio (95% CI)	Lean Sport Athletes	Non-Lean Sport Athletes	*p*-Value	Odds Ratio (95% CI)
RE	24.6% (207/841)	24.7% (54/219)	24.6% (153/622)	0.986	1.00 (0.70–1.43)	23.5% (116/494)	26.2% (91/347)	0.363	0.86 (0.63–1.19)	**26.9% (146/542)**	**20.4%** **(61/299)**	**0.035**	**1.44** **(1.03–2.02)**
ED	18.4% (155/843)	18.1% (40/221)	18.5% (115/622)	0.898	0.97 (0.66–1.45)	**14.6% (72/494)**	**23.8% (83/349)**	**0.001**	**0.55** **(0.39–0.78)**	**20.6% (112/543)**	**14.3%** **(43/300)**	**0.024**	**1.55** **(1.06–2.28)**
MD	31.6% (160/506)	27.0% (33/122)	33.1% (127/384)	0.213	0.75 (0.48–1.18)	**35.0% (111/317)**	**25.9% (49/189)**	**0.033**	**1.54** **(1.03–2.29)**	**35.6% (115/323)**	**24.6%** **(45/183)**	**0.010**	**1.70** **(1.13–2.55)**
PA	15.5% (129/832)	11.9% (26/219)	16.8% (103/613)	0.084	0.67 (0.42–1.06)	15.7% (77/490)	15.2% (52/342)	0.842	1.04 (0.71–1.52)	17.3% (93/538)	12.2% (36/294)	0.055	1.50 (0.99–2.27)
Injury	55.3% (468/846)	50.7% (112/221)	57.0% (356/625)	0.106	0.78 (0.57–1.06)	55.6% (276/496)	54.9% (192/350)	0.820	1.03 (0.78–1.36)	**50.8% (277/545)**	**63.5% (191/301)**	**<0.001**	**0.60** **(0.45–0.79)**
≥22 missed days ^a^	30.8% (143/464)	29.7% (33/111)	31.2% (110/353)	0.776	0.94 (0.59–1.49)	32.2% (88/273)	28.8% (55/191)	0.430	1.18 (0.79–1.760)	29.6% (81/274)	32.6% (62/190)	0.481	0.87 (0.58–1.29)

^a^ From injured athletes. CI = confidence interval. Statistically significant *p*-values are indicated in bold.

**Table 2:** Frequencies and proportions of athletes with one or more injury during the preceding year and athletes with ≥22 missed participation days due to injuries during the preceding year, along with crude odds ratios by restrictive eating (RE), eating disorders (ED), and menstrual dysfunction (MD).

One or More Injury					≥22 Missed Participation Days Due to Injuries ^a^				
Variable	*n*	Proportion (%)	OR	95% CI	Variable	*n*	Proportion (%)	OR	95% CI
No RE	339	53.5	1.00	Reference	No RE	104	30.8	1.00	Reference
RE	128	**61.8**	**1.41**	**1.02–1.94**	RE	39	31.2	1.02	0.66–1.59
No ED	362	52.6	1.00	Reference	No ED	117	32.6	1.00	Reference
ED	105	**67.7**	**1.89**	**1.31–2.73**	ED	26	25.0	0.69	0.42–1.13
No MD	187	54.0	1.00	Reference	No MD	50	27.3	1.00	Reference
MD	87	54.4	1.01	0.70–1.48	MD	35	**40.2**	**1.79**	**1.05–3.07**

^a^ Only injured participants were included in this analysis. OR = odds ratio; CI = confidence interval. Statistically significant differences are indicated in bold.

**Table 3:** Adjusted odds ratios for one or more injury during the preceding year and ≥22 missed training days due to injury during the preceding year by restrictive eating (RE), current or past eating disorder (ED), and menstrual dysfunction (MD).

One or More Injury			≥22 Missed Participation Days due to Injuries ^a^		
Risk Factor	OR ^b^	95% CI	Risk Factor	OR ^b^	95% CI
RE	**1.50**	**1.05–2.14**	RE	0.92	0.57–1.49
ED	**2.29**	**1.52–3.44**	ED	0.64	0.38–1.08
MD	1.10	0.72–1.67	MD	1.63	0.91–2.91

^a^ Only injured participants were included in this analysis; ^b^ Adjusted for BMI, training hours in the preceding year, and age. OR = odds ratio; CI = confidence interval. Statistically significant differences are indicated in bold.

## Data Availability

Data presented in this study are available on reasonable request from the corresponding author.

## Supplementary Materials

The following are available online at https://www.mdpi.com/article/10.3390/nu13093275/s1: Table S1. List of sports reported by the athletes; Table S2. Characteristics of the participants and comparisons between the athletes classified by their competition level, age, and type of sport.
