# Supplementary material for: Self-Reported Restrictive Eating, Eating Disorders, Menstrual Dysfunction, and Injuries in Athletes Competing at Different Levels and Sports
Source: Nutrients. 2021 Sep 19;13(9):3275. doi: 10.3390/nu13093275 (PMC8470308; doi:10.3390/nu13093275)
Supplement: Supplementary file 1 [file nutrients-13-03275-s001.zip › nutrients-1364574-supplementary.pdf]

Supplemental material

Table S1. List of sports reported by the athletes (number of athletes in parentheses).

Lean sports (n = 545, 64.4% of the whole sample)	Non-lean sports (n = 301, 35.6% of the whole sample)
Biathlon (1)	Agility (1)
Brazilian jiu-jitsu (10)	Alpine skiing (2)
Cheerleading (43)	American football (22)
Climbing (2)	Badminton (3)
Cross-country skiing (42)	Bandy (7)
Cycling (8)	Basketball (2)
Dancing (23)	Beach volley (3)
Diving (2)	Crossfit (17)
Figure skating (51)	Disc golf (14)
Fitness (11)	Fencing (1)
Gymnastic (21)	Finnish baseball (10)
Hurdling (3)	Flag football (2)
Judo (7)	Floorball (36)
Kickboxing (1)	Futsal (2)
Middle- and long-distance running (34)	Handball (6)
Mixed martial arts (1)	Horseback riding (5)
Mushing (2)	Ice hockey (33)
Orienteering (30)	Motorsport (2)
Powerlifting (9)	Ringette (15)
Race walking (2)	Roller derby (1)
Rowing (4)	Rugby (2)
Skating (1)	Sailing (2)
Steeplechase (1)	Shooting (1)
Street workout (1)	Snowboarding (1)
Strongman (2)	Soccer (78)
Swimming (115)	Speed skating (3)
Swimrun (1)	Sprint (3)
Synchronized swimming (14)	Taido (1)
Taekwondo (6)	Tennis (1)
Thai boxing (1)	Ultimate (1)
Track and field (antigravitation sports) (17)	Underwater rugby (1)
Triathlon (24)	Volleyball (17)
Weightlifting (51)	Water polo (6)
Wrestling (4)	

Table S2. Characteristics of the participants and comparisons between the athletes classified by their competition level, age, and type of sport.

	All participants	Non-elite athletes	Elite athletes	Younger athletes	Older athletes	Lean sport athletes	Non-lean sport athletes
Age (years), mean (SD)	24.3 (7.5) (n = 846)	27.0 (8.6) (n = 221)	23.3 (6.8) (n = 625)	19.0 (2.6) (n = 496)	31.8 (5.4) (n = 350)	24.3 (5.8) (n = 545)	24.4 (6.9) (n = 301)
Height (cm), mean (SD)	167.8 (6.5) (n = 846)	167.3 (5.9) (n = 221)	168.0 (6.6) (n = 625)	168.2 (6.5) (n = 496)	167.3 (6.4) (n = 350)	**167.4 (6.4)*** (n = 545)	**168.7 (6.6)*** (n = 301)
Weight (kg), median (IQR)	64.0 (58–70) (n = 841)	64.0 (58–70) (n = 219)	64.0 (58–70) (n = 622)	**63.0 (57–68)*** (n = 491)	**65.0 (59–72)*** (n = 350)	**63.0 (57–68)*** (n = 544)	**65.0 (61–73)*** (n = 297)
BMI (kg/m^2), median (IQR)	22.6 (20.8–24.3) (n = 841)	22.8 (21.0–25.1) (n = 219)	22.5 (20.8–24.2) (n = 622)	**22.5 (20.7–23.8)*** (n = 491)	**23.2 (21.1–25.6)*** (n = 350)	**22.3 (20.6–24.1)*** (n = 544)	**23.2 (21.6–25.4)*** (n = 297)
% BMI < $18.5 kg/m^2$	2.6 (22) (n = 841)	2.23 (5) (n = 219)	2.7 (17) (n = 622)	3.1 (15) (n = 491)	2.0 (7) (n = 350)	3.3 (18) (n = 544)	1.3 (4) (n = 297)
Training hours[a], median (IQR)	550 (384–728) (n = 726)	**484 (250–574)*** (n = 189)	**600 (450–786)*** (n = 537)	**624 (436–850)*** (n = 413)	**467 (300–600)*** (n = 313)	540 (390–730) (n = 484)	561 (380–728) (n = 242)

[a] During the preceding year.
* Statistically significant difference ($p < 0.05$) compared with the comparison group.
SD = standard deviation; IQR = interquartile range
